# Regulation of uterine function during estrous cycle, anestrus phase and pregnancy by steroids in red deer (*Cervus elaphus *L.)

**DOI:** 10.1038/s41598-021-99601-5

**Published:** 2021-10-11

**Authors:** Angelika M. Kotlarczyk, Martyna Grzyb, Anna J. Korzekwa

**Affiliations:** Department of Biodiversity Protection, Institute of Animal, Reproduction and Food Research of Polish Academy of Sciences (IAR&FR PAS), Tuwima 10 Str., 10-748 Olsztyn, Poland

**Keywords:** Biotechnology, Developmental biology, Molecular biology, Endocrinology

## Abstract

Steroid synthesis and production in ruminant uterus is not obvious, especially in seasonally reproduced. We compared steroid production by investigating enzymes involved in red deer uterine steroid metabolism in reproductive seasons. Blood and uteri (endometrium and myometrium) were collected *post mortem* from hinds on 4th day (N = 8), 13th day of the cycle (N = 8), anestrus (N = 8) and pregnancy (N = 8). The expression of cytochrome P450 aromatase (P450), 3 -beta-hydroxysteroid dehydrogenase (3β-HSD), 17 -beta-hydroxysteroid dehydrogenase (17β-HSD), aldo–keto reductase family 1 C1 (AKR1C1), estrogen receptor alpha (ERα), and progesterone receptors (PRs), were analyzed using real-time-PCR and Western Blotting. Plasma samples were assayed for 17-beta-estradiol (E2), progesterone (P4), luteinizing hormone (LH), follicle-stimulating hormone (FSH), and testosterone (T4) concentrations by EIA. Hinds at the beginning of the estrous cycle, mainly in endometrium, were characterized by a high mRNA expression of 3β-HSD, AKR1C1, PRs and ERα, contrary to the expression in myometrium during pregnancy (*P* < 0.05). For P4, E2, and FSH, concentration was the highest during the 13th day of the estrous cycle (*P* < 0.05). Uterine steroid production and output in hinds as a representative seasonally reproduced ruminant occurred mainly during the estrous cycle and sustained in anestrus.

## Introduction

There is almost any information concerning steroidogenesis in uterus of ruminants, especially in those seasonally reproduced. Red deer belongs to short-day ruminants, whose breeding season is associated with photoperiod and a shortening day^1^. The reproductive cycle begins at the end of September and lasts until the end of November in temperate climates^2–5^. Most studies on the female reproductive system of ruminants seasonally reproduced focus on ovarian activity^6–8^. There exist a lack of knowledge that would clearly describe the synthesis and action of steroids in the uterus in seasonally reproduced ruminants and its control by the pituitary, which red deer represents.

In the uterus and ovary, progesterone (P4) plays key physiological roles under the control of the pituitary hormones: luteinizing hormone (LH) and follicle-stimulating hormone (FSH)^9^ and it affects the release of mature oocytes, supports implantation, maintains of pregnancy, and inhibits contractions of the myometrium^10^. During pregnancy, this steroid is synthesized by the corpus luteum, however, in the further stages of pregnancy, the placenta produces it in most mammal species, such as cow^11^, pig^12^, roe deer^13^, and human^14^. The physiological effect of P4 is carried out by a genomic mechanism via nuclear P4 receptors (PRs) and a non-genomic mechanism. The nuclear PRs occur in two main isoforms: isoform A (PRA) and isoform B (PRB), transcribed from the same gene but under the influence of two different promoters^15^.

In the endometrial follicular phase, estrogens induce the proliferation of endometrial epithelium. The target tissue for P4 and estrogen (E2) is the endometrium, which undergoes biochemical and morphological changes. These hormones’ effects on endometrium function are mediated by estrogen receptors (ERs)^16^. There are two primary estrogen receptors, which are ERα and ERβ. They are ligand-activated transcription factors and mainly represent response genomic action mechanism. The primary site of the ERα expression throughout the female reproductive tract is the uterus^17^, and we decided to define only the expression of this estrogen receptor isoform.

The enzymes involved in the synthesis of steroids belong to the hydroxysteroid dehydrogenases (HSDs) or cytochrome P450 monooxygenases (CYPs). Hydroxysteroid dehydrogenases include 3β-HSD and 17β-HSD. The CYP enzymes include CYP11A1 which cleaves the cholesterol side chain, bifunctional CYP17A1 and CYP19A1 as aromatase^18^. The cytochromes P450 constitute a large family of enzymes and play a crucial role in converting exogenous and endogenous molecules^19^. The first stage is the transfer of cholesterol from outer to inner mitochondrial membranes. This process is controlled by the steroidogenic acute regulatory protein (StAR)^20^. Then, the side-cleaving enzyme (P450scc) converts cholesterol to pregnenolone^21^. The activity of the 3β-HSD enzyme metabolizes pregnenolone to P4. The biological activity of steroid hormones is also regulated by 17β-HSD. The role of 17β-HSD is the conversion of estrone (E1) and E2, and modulation of the tissue concentrations of bioactive E2. In target tissues, 17β-HSD isoenzymes regulate the concentration of 17β-keto and 17β-hydroxy forms of estrogens and androgens.

The aldo–keto reductase family 1 member C1 (AKR1C1) belongs to a family of NADPH-dependent reductases that degrades P4. This enzyme with 20α-hydroxysteroid dehydrogenase (20α-HSD) activity forms progestin and plays a crucial role in maintaining pregnancy. A wide range of substrates, including steroid hormones, endogenous prostaglandins, and carbohydrates, are converted by AKR1C1^22^. 20α-hydroxysteroid dehydrogenase is expressed in several tissues of the reproductive tract, such as the ovary, uterus, cervical canal, and placenta, such as in rats, goats, humans, and pigs^23^.

One conserved function of steroid hormone receptors is that they autoregulate the expression of their genes. Estrogens up-regulate both ER and PR gene expressions in uteri of all mammalian species^24^. The correlation between enzymes involved in steroid metabolism, receptivity, and peripheral concentration in the uterus has not been described in the red deer yet in different stages of reproductive seasons. We hypothesized that in hinds dependently on the reproductive season in the uterus: (1) P450, AKR1C1, 3β-HSD, and 17β-HSD are involved in steroids regulation differently; (2) receptivity of P4 and E2 for ovarian steroids is variable, (3) concentration of LH, FSH, and basic ovarian steroids (P4, E2, and T4) correlate with the expression of mentioned enzymes dependently to the reproductive status.

Given the photoperiod-dependent readiness to reproduce in the red deer and the poorly understood processes of steroidogenesis regulation and the action of steroids in the uterus this study aimed to determine: (i) the mRNA and the protein expressions of P450, 3β-HSD, 17β-HSD, ERα, PRs, and AKR1C1 involved in steroid metabolism and (ii) concentration of E2, P4, LH, FSH and T4 in plasma during the estrous cycle (follicular and luteal stages), the anestrus, and pregnancy.

## Results

### P450, 3β-HSD, 17β-HSD, AKR1C1 enzymes engagement in the estrous cycle, anestrus, and pregnancy

#### mRNA and protein expressions in endometrium

**Figure 1:**
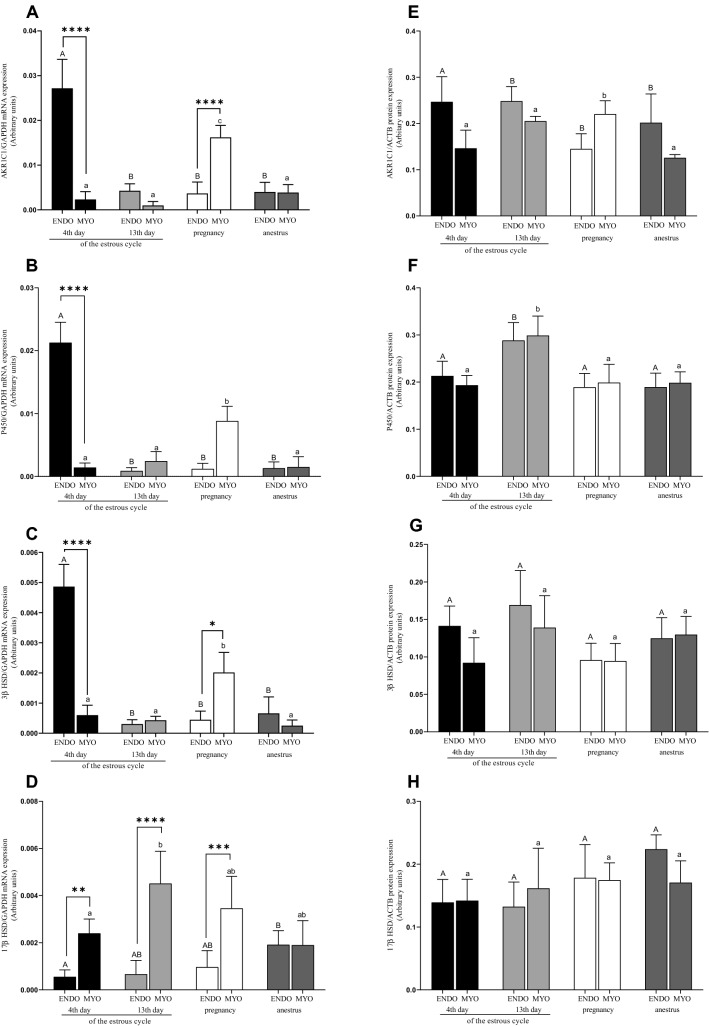
mRNA and protein expression of AKR1C1 (**A**, **E**), P450 (**B**, **F**), 3β-HSD (**C**, **G**) and 17β-HSD (**D**, **H**) in uterine tissues (endometrium and myometrium) on 4th and 13th day of estrous cycle, in pregnancy and anestrus phase. Data were normalized against GAPDH for mRNA expression and against β-actin (ACTB) for proteins expression. Each bar represents one experimental group with SEM. Statistical differences were analyzed by two-way analysis (ANOVA) of variance followed by the Bonferroni post hoc test using GraphPad PRISM (Version 8.3.0). The lowest statistical significance was *P* < 0.05. Asterisks indicate statistical differences between endometrium and myometrium (*P < 0.1; **P < 0.01; ***P < 0.001; ****P < 0.0001). Different letters indicate statistical differences (*P* < 0.05) between the experimental groups throughout endometrium (A, B) and myometrium (a–b) respectively.

The mRNA expression for AKR1C1, P450, and 3β-HSD was higher on the 4th day of the estrous cycle than on the 13th day of the estrous cycle, pregnancy and the anestrus groups (*P* < 0.05; Fig. 1A–C). For 17β-HSD, the mRNA expression increased during the anestrus compared with the 4th day of the estrous cycle (*P* < 0.05; Fig. 1D).

The protein expression for AKR1C1 was the highest on 4th day of the estrous cycle and differed from 13th day of cycle, the anestrus phase and pregnancy (*P* < 0.05; Fig. 1E). P450 protein expression was higher in the endometrium on the 13th day than other experimental groups (*P* < 0.05; Fig. 1F). 3β-HSD and 17β-HSD protein expression was on the similar level throughout the examined experimental periods (*P* > 0.05; Fig. 1G,H).

#### mRNA and protein expressions in myometrium

For AKR1C1, P450 and 3β-HSD, the highest mRNA expression occurred in pregnancy and increased compared with the 4th, 13th day of the estrous cycle, and the anestrus (*P* < 0.05; Fig. 1A–C), whereas 17β-HSD was up-regulated on 13th day of the cycle comparing to 4th day of the cycle (*P* < 0.05; Fig. 1D).

The highest AKR1C1 protein expression was observed in pregnancy and differed from all examined groups (*P* < 0.05; Fig. 1E). However, the P450 protein expression was the highest on the 13th day of the estrous cycle and differed from the anestrus, pregnancy, and 4th day of the estrous phase (*P* < 0.05; Fig. 1F). 3β-HSD and 17β-HSD protein expression was on the similar level throughout the examined experimental periods (*P* > 0.05; Fig. 1G,H).

#### Interaction between expression in endometrium and myometrium

Interaction in mRNA expression occurred for three of them (AKR1C1, P450, 3β-HSD) and was higher in endometrium than in myometrium on 4th day of estrous cycle (*P* < 0.0001; Fig. 1A–C). In addition for AKR1C1 mRNA expression was higher in myometrium in pregnancy group than on 4th day of estrous cycle (*P* < 0.0001; Fig. 1A). For 17β-HSD, the interaction occurred between all tested groups except the anestrus and was higher in myometrium for all three of them. However, the highest mRNA expression was on the 13th day of the estrous cycle (*P* < 0.0001), lower in pregnancy group (*P* < 0.001) and the lowest on 4th day of the estrous cycle in myometrium (*P* < 0.01; Fig. 1D).

### Receptivity of uterus for P4 and E2 during the estrous cycle, anestrus, and pregnancy

#### mRNA and protein expressions in endometrium

**Figure 2:**
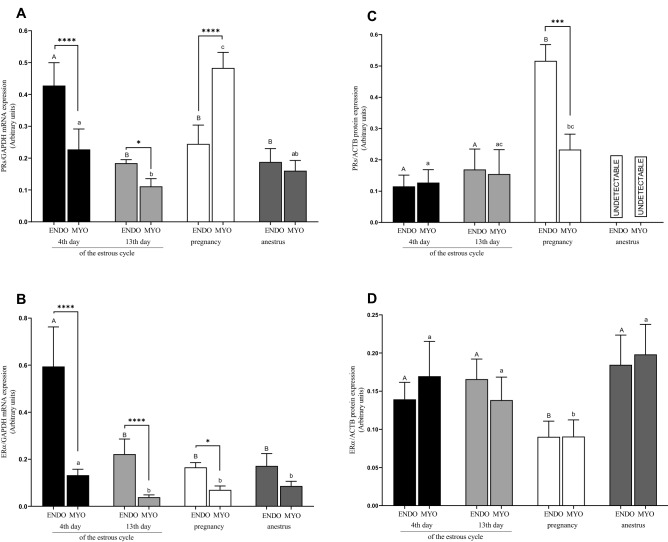
mRNA and protein expression of PRs (**A**, **C**) and ERα (**B**, **D**) in uterine tissues (endometrium and myometrium) on 4th and 13th day of estrous cycle, in pregnancy and anestrus phase. Data were normalized against β-actin (ACTB) for proteins expression and against GAPDH for mRNA expression. Each bar represents one experimental group with SEM. Statistical differences were analyzed by two-way analysis of variance (ANOVA) followed by the Bonferroni post hoc test using GraphPad PRISM (Version 8.3.0). The lowest statistical significance was *P* < 0.05. Asterisks indicate statistical differences between endometrium and myometrium (*P < 0.1; ***P < 0.001; ****P < 0.0001). Different letters indicate statistical differences (*P* < 0.05) between the experimental groups throughout endometrium (A, B) and myometrium (a–c) respectively.

Compared with the anestrus, pregnancy, and 13th day, PRs and ERα mRNA expression increased on the 4th day of the estrous cycle in the endometrium (*P* < 0.05; Fig. 2A,B). The highest PRs protein expression was in pregnant hinds and differed from the 4th and 13th day of the estrous cycle (*P* < 0.05; Fig. 2C). ERα protein expression was the lowest in pregnancy, compared with the 4th, and 13th day of the estrous cycle, and the anestrus in the endometrium (*P* < 0.05; Fig. 2D).

#### mRNA and protein expression in myometrium

An increase in the mRNA expression was observed for PRs in the myometrium during pregnancy compared with other groups and PRs mRNA expression on 4th day of cycle was higher than on 13th day of the cycle and anestrus (*P* < 0.05; Fig. 2A). The mRNA expression in myometrium for ERα was lower on the 13th day of the estrous cycle, pregnancy, and the anestrus compared with the 4th day of the estrous cycle (*P* < 0.05; Fig. 2B). PRs protein expression in pregnancy was up-regulated compared with the 4th day of the estrous cycle (*P* < 0.05; Fig. 2C). ERα protein expression was higher on the 4th and, 13th day of the estrous cycle and the anestrus than in pregnancy (*P* < 0.05; Fig. 2D).

#### Interaction between expression in endometrium and myometrium

For PRs mRNA expression, an interaction between endometrium and myometrium was exhibited during the 4th and 13th day of the estrous cycle (*P* < 0.05; Fig. 2A) and during pregnancy (*P* < 0.0001; Fig. 2A). In the case of ERα, the mRNA expression in the estrous cycle (4th and 13th day) confirmed the interaction between the tissues examined and the expression was up-regulated in the endometrium than myometrium (*P* < 0.0001; Fig. 2B). Additionally, an interaction was exhibited during pregnancy and the expression was higher in the endometrium (*P* < 0.05; Fig. 2B). PRs protein expression in the pregnancy in the endometrium was higher than in the myometrium (*P* < 0.001; Fig. 2C).

### Plasma concentration of P4, E2, T4 LH, and FSH

**Figure 3:**
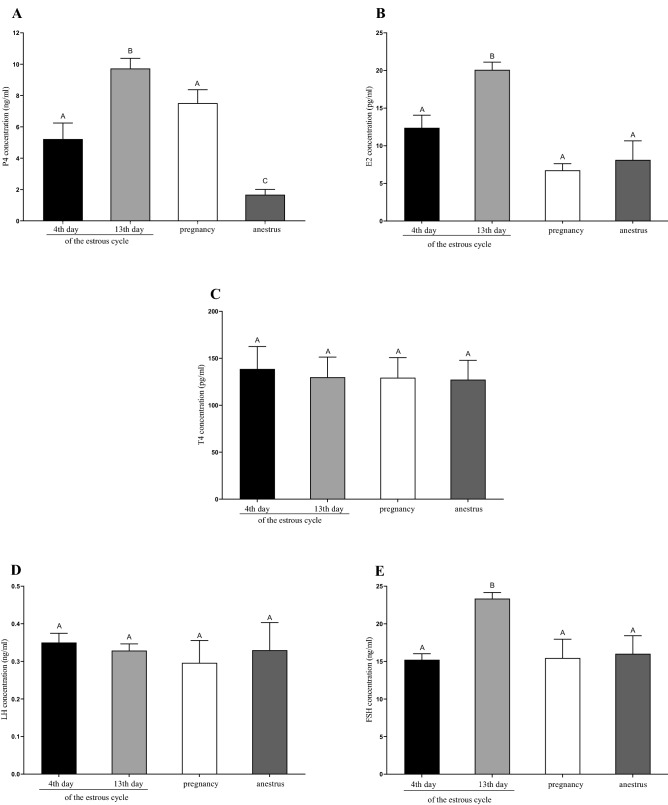
Plasma concentration of P4, E2, T4 LH, and FSH in the blood samples collected on 4th and 13th day of estrous cycle, in pregnancy and anestrus phase. Plasma concentrations were estimated using ELISA kits, according to the manufacturer’s instructions. Statistical differences were analyzed by one-way ANOVA, followed by Tukey’s test, using GraphPad PRISM (Version 8.3.0). The lowest statistical significance pointed by different letters (A–C) was *P* < 0.05.

The plasma P4, E2, and FSH levels were higher on the 13th day of the estrous cycle than other groups (*P* < 0.05; Fig. 3A,B,E). The level of P4 was the nearest in anestrus comparing to other experimental periods (*P* < 0.05; Fig. 3A). T4 and LH concentrations were on a similar level, and no significant differences were observed (*P* > 0.05, Fig. 3C,D).

## Discussion

Most of our understanding of the steroid action in uterus comes from primate research^24^, whereas knowledge about steroid pathways regulating seasonal reproductive cycles in red deer is weak^25,26,28^ and comes mainly from research using sheep and goats^29,30^. For the first time, using red deer females as an experimental model, we demonstrated that the steroid pathways regulation is different and varies with the reproductive seasons. The breeding season and anestrus were characterized by maximum endometrial mRNA expression of 3β-HSD, AKR1C1, PRs, ERα, whereas pregnancy by the highest mRNA myometrial expression for P450, 3β-HSD, AKR1C1, 17β-HSD and PRs. The fluctuations in protein expression were not so spectacular but occurred mainly in myometrium for AKR1C1 during pregnancy and for 3β-HSD in anestrus. Changes in reproductive status may result from steroid-feedback mechanisms found in sheep and goats^30^.

### P450, 3β-HSD, 17β-HSD, AKR1C1 enzymes engagement in the estrous cycle, the anestrus and pregnancy

According to Rękawiecki et al.^31^, the mRNA expression and the protein for main enzymes involved in steroidogenesis (cytochrome P450scc and 3β-HSD) in the bovine uterus at the beginning of the estrous cycle are high. However, the opposite effect occurred in bovine endometrium on 17–20 days of the estrous cycle. These results suggested that P450 and 3β-HSD expression were reduced in the later estrous cycle phases^32^. We obtained similar results in the hind uterus. At the early luteal stage—4th day of the estrous cycle in the endometrium, the mRNA expression increased for P450 and 3β-HSD compared with the 13th day of the estrous cycle and other experimental groups. However, P450 protein expression was up-regulated on the 13th day of the estrous cycle both in the endometrium and in the myometrium. No much information is available about the expression of 17β-HSD in ruminants’ uterus. However, in pigs it has been well understood. Wojciechowicz et al.^33^ observed a decrease of 17β-HSD mRNA and protein expression in the pig endometrium on 15–16 days of pregnancy and the ovary on 15–16 days of luteolysis. We received the increase of 17β-HSD mRNA expression in myometrium which may indicate that the process of steroid inactivation occurs during this periods in myometrium, however such up-regulation was not received on the level of protein expression.

In turn, Naidansuren et al.^13^ examined the mRNA and protein expression for 20α-HSD in the placenta, ovary, and uterus. Because they reported that in the placenta, the expression mRNA and protein for 20α-HSD were higher than in the ovary and uterus, they suggested that deer placenta plays a crucial role in P4 regulation on 30, 60 and 70 days of pregnancy. However, according to Kim et al.^34^, during early pregnancy bovine ovary contributes significantly to steroid hormone regulation because it is the primary source of 20α-HSD mRNA and protein production, although the placenta and endometrium synthesize it as well^34^. This study confirmed 20α-HSD mRNA and the protein expression in the deer uterus as its biological activity in AKR1C1. Therefore, the mRNA expression and the protein were lower in pregnancy and the estrous cycle than in the anestrus in endometrium on the contrary to myometrium, where its expression was the highest during pregnancy. Explanation of such results in endometrium may be the readiness of red deer uterus for the resumption of steroids production necessary for the beginning of the reproductive season and the estrous phase. Whereas production of P4 by myometrial AKR1C1 may support the pregnancy development.

### Receptivity of uterus for P4 and E2 during the estrous cycle, anestrus and pregnancy

In the uterus of sheep and sika deer distribution of PRs and ERα differed between endometrium and myometrium. In sheep, staining of ERα decreased on the 11th day of the estrous cycle in uterine tissue and remained weak during pregnancy; although in sika deer, staining of ERα was low during the estrous cycle in the endometrium. High staining was detected during the early pregnancy of sika deer (20–25 days) for PRs in myometrium similar in sheep and cattle^35^. Similar results obtained by Garcίa-Palencia et al.^36^, showed differences in expressions of ERα and PRs in the uterus of sheep after the estrous cycle was synchronized by prostaglandin analogue or by treatment with progestagens. The sheep treated with progestagens showed a decreased ER expression in the superficial uterine compartment (luminal epithelium, glands, and stroma) and myometrium and in the PR expression in all the uterine cell compartments except the myometrium. The reduction of both receptors’ expression in the superficial compartments could compromise embryonic implantation due to the significance these steroid receptors’ in maternal identification during pregnancy. According to Duan et al.^30^, ERα mRNA expression level was high in the sheep endometrium and myometrium and for PRs’ expression in myometrium during the estrous cycle. However, in the estrous cycle, PRs’ protein expression in the myometrium was significantly low. These results suggest that both receptors are essential for sheep reproductive function in the uterus, but its expression is changeable in uterus tissues^30^. It is known that estrogens during the follicular phase up-regulate both PRs and ERα in the uterus of most mammalian and P4 during the luteal phase down-regulates them; it should be taken into account that mRNA expressions do not always reflect protein concentration^37^. Our research results in which the mRNA expression for PRs was the highest in pregnancy in myometrium conformed other studies. Protein expression for PRs was also the highest during pregnancy, however, in both uterine tissues. Rekawiecki et al.^38^ found that high protein levels for both PRs isoforms at the beginning of the protein expression were the highest during the anestrus phase in both (endometrium and myometrium) tissues examined. In turn, the ERα mRNA expression was the highest on the 4th day of the estrous cycle in the endometrium and the nearest its protein expression was received during pregnancy. Such results indicate that especially during the pregnancy development uterus doesn`t need additional source of estrogens.

### Plasma concentration of P4, E2, T4, LH and FSH

An essential finding reported here was the marked seasonal variation of LH, FSH, P4, E2, and T4 concentrations in hinds’ blood plasma as described before^39^. Our results have clearly shown that LH concentration in hinds’ plasma was at a similar level during all analyzed seasons, but we did not analyze the preovulatory outflow period. Nevertheless, our results differ from the results obtained by Li et al.^9^. In anestrus sheep, the concentration of LH in the serum was lower than during the reproductive season. Opposite LH concentration results during anestrus obtained by Meikle and Fisher^28^ in ovariectomized red female deer. The anestrus was characterized by a maximum suppression of LH and minimum suppression of breeding season. These changes were similar to the intact herdmates. This agrees with work using sheep and goats and indicates a change in the steroid-feedback mechanism and changes in reproductive status. Yuthasastrakosol et al.^40^ noted the high LH levels in anestrus in ewes, and they suggested an association of these values with the increase in P4 levels. Low levels of P4 during anestrus in goats and high levels of P4 in the luteal phase in sheep, during the middle of reproductive season, were observed by Thimonier^39^. In turn, Li et al.^9^ did not observe a luteal P4 peak in sheep during anestrus. In comparison, our results showed a significant increase in P4 concentrations during the 13th day of the estrous cycle, while in the anestrus phase, this hormone’s level was the lowest. On the 13th day of the estrous cycle, we noted the highest concentrations for FSH and E2 in the hind plasma. In cattle, low P4 allows FSH to increase from the 16th day of the cycle and the E2 concentration decreases after that, along with the regression of subordinate follicles^41^. In pregnant cows, there are few reports on the peripheral plasma levels of androgens. According to Gaiani et al.^42^, plasma T4 values increased throughout pregnancy. Kanchev et al.^43^ reported an increase in plasma T4 concentrations at the onset of luteolysis in non-pregnant cows. In turn, Herriman et al.^44^ compared measurements of the plasma concentrations of T4 during the estrous cycle of ewes and heifers. The maximum concentration of T4 was similar in both species and occurred at the onset of luteolysis; in the ewe, the peak concentration of plasma was significantly greater than the concentrations of T4 measured during the remainder of the estrous cycle. According to our research, the T4 concentration in the female red deer was at a similar level in all tested phases, with the tendency to decrease in the anestrus.

## Conclusion

The mRNA expression for all examined factors, except 17β-HSD, was the highest on the 4th day of the estrous cycle in the endometrium. Receptivity for P4 is changeable, and PRs mRNA expression is up-regulated in the endometrium during the examined days of the estrous cycle but not during the pregnancy. Whereas in pregnancy, PRs protein expression is increased in the endometrium because it is responsible for maintaining pregnancy. PRs mRNA expression is elevated in pregnancy as if it additionally played a supporting role in its development. The profile of mRNA, protein expression and immunolocalization of PRS was described by Kowalik et al.^45^ in uterine endometrium and myometrium in cows. The authors pointed that in both uterine layers, those receptors are expressed. Moreover the localization of PRS in the endothelial cells of blood vessels in the bovine uterus, according to the mentioned authors, suggests that P4 affects blood flow in this organ through mPRs^45^. Receptivity for ERα and expression of P450 is up-regulated only at the beginning of the cycle, probably for enhancement of follicles' development during the estrous cycle. In the case of hormones concentration, the highest values were on the 13th day of the estrous cycle for P4, E2, and FSH, whereas for T4 and LH, no differences we found between the examined seasons, and further studies are necessary for the detailed recognition of hormone production with more examined days of the estrous cycle and pregnancy in hinds. However, it is evident that steroid regulation in hinds is variable, dependently on the reproductive status, and prevails in the endometrium over myometrium and differ from non-seasonal ruminants. Knowledge of the molecular mechanisms by which steroid hormones regulate gene expression for their receptors is critical for understanding steroid hormone actions during the estrous cycle, anestrus, and pregnancy in the uterus in the female red deer as an example of seasonally reproduced ruminants.

## Material and methods

### Sample collection

The experimental material (blood samples collected from the heard and uteri) was collected *post mortem* 15–20 min after the shot (3rd–5th of January 2019, the third month of pregnancy) from wild hinds in the Strzałowo Forest District (North-East of Poland; N = 8) during hunting season 2018/2019 (hunting license: ZG7521-3/2018/2019) and from breeding females from a farm in Rudzie near Gołdap (North-East of Poland; N = 24). A total number of thirty two (N = 32) 3–4-year-old hinds were evaluated, and the age was confirmed, according to Korzekwa et al.^25^ All veterinary procedures were conducted after receiving the agreement of the Local Ethical Committee for Animal Experiments in Olsztyn (Poland, Agreement Number 7/2019). All experiments were performed in accordance with ARRIVE guidelines the study was conducted in accordance with relevant guidelines and regulations and approved by Welfcare Ethics Committee in the Institute of Animal Reproduction and Food Research of Polish Academy of Sciences in Olsztyn (Poland**).** Moreover informed consent was obtained from the farm owner for the collection of samples.

The first experimental group (4th day of the estrous cycle) representing the follicular phase (N = 8), and the second experimental group (13th day of the estrous cycle) representing the luteal phase (N = 8) were obtained on the 17th and 23rd of September 2019 after pharmacological synchronization on the farm in Rudzie. The estrus and ovulation induction hinds during the estrous cycle was performed by applying a double controlled internal drug-release (CIDR) insert (1.38 g of P4; Pfizer Animal Health, New York, US), using a 12-day regimen of intravaginal CIDR devices. For better synchronization, the device was replaced after 7 days to maintain the luteal concentration of P4 until the end of the treatment period. Additionally, 200 IU of human chorionic gonadotropin (hCG; Folligon, Intervet, International B.V., Boxmeer, Holland) was injected intramuscularly on day 12. The estrus was observed 54–56 h after the second CIDR insert removal in the hinds. The day of the estrous cycle was evaluated by macroscopically observing the ovaries and uterus and confirmed by determining E2 and P4 levels in the blood plasma using an Enzyme-Linked Immunosorbent Assay (ELISA)^25,26^. The reasons for culling animals from the herd on the farm were for economic considerations and herd renewal.

The third group (hinds in the anestrus) were collected from non-pregnant females on the 10th of May, 2019 (N = 8) on the farm in Rudzie. The last experimental group (pregnant hinds) was possessed between the 2nd and 4th of January 2019 (N = 8) from wild individuals in Strzałowo Forestry. Pregnacy was confirmed by the presence of embryo and PAGs concentration determination examined by EIA according to Korzekwa et al.^27^. The uterine horn tissue was immediately placed on ice and transported in liquid nitrogen to the laboratory, where it was subsequently stored at − 80 °C for further procedures. Plasma samples were collected by jugular venipuncture (EDTA-loaded vacuum tubes). Samples were held in ice until centrifugation at 3000 g at 4 °C for 10 min after which the plasma was placed on ice and transported to the laboratory within 30 min and stored at − 20 °C until assayed by ELISA.

### Experimental procedure

P450, 3β-HSD, 17β-HSD, and AKR1C1 enzymes engagement in the estrous cycle, anestrus, and pregnancy was determined in the uterine endometrial and myometrial tissue by real-time RT -PCR and Western Blotting.

Receptivity of uterus for P4 and E2 during the estrous cycle, anestrus and pregnancy was determined in the endometrium and myometrium tissue by real-time RT -PCR and Western Blotting.

Concentration of LH, FSH, P4, E2 and T4 involved in enzymes metabolism was assayed by ELISA.

### Determinations

#### Total RNA isolation and reverse transcription

Uterine tissue was first homogenized in liquid nitrogen, and then total RNA was isolated with TRI-Reagent (Sigma-Aldrich, Darmstadt, Germany, T9424), according to the manufacturer's instructions. After extraction, the purity and RNA concentration was assessed with NanoDrop 1000 spectrophotometer (Thermo Fisher Scientific, Wilmington, DE). The wavelength ratio for all samples neared 2.0 for 260/280 nm and ranged between 1.8 and 2.2 for 260/230 nm. RNA, 1 μg was calculated based on spectrophotometric measurement and then reverse—transcribed using the High Capacity cDNA Reverse Transcription Kit (Applied Biosystems, Cheshire, UK, 4368813), which contained a MultiScribe™ reverse transcriptase with random primers, RNase Inhibitor, MgCl_2,_ dNTP mixture, and Nuclease—free H_2_O. The samples were incubated at 25 °C for 10 min followed by 37 °C for 2 h. Finally, to inactivate the reverse transcriptase, the temperature was increased to 85 °C for 5 min. Obtained cDNA was kept at − 20 °C until further analysis.

### Real-time PCR

The mRNA expression of P450, 3β-HSD, 17β-HSD, AKR1C1, PRs, and ERα enzymes in endometrium and myometrium was analyzed by RT-PCR using Applied Biosystems Real-Time 7900 system (Applied Biosystems, Cheshire, UK), with SensiFAST SYBR Hi-ROX Kit (Bioline Reagents, London, UK, BIO-92002) according to the manufacturer’s instructions. Gene-specific primer sequences of P450, 3β-HSD, 17β-HSD, AKR1C1, PRs, and ERα genes were designed using Primer Express Software v.3. The final PCR mix (10 µL) contained 3 µl of reverse-transcribed cDNA (15 ng), 5 µL of SensiFAST SYBR Hi-ROX Mix (SYBR Green and 3 Mm of MgCl_2_) and 0.2 µL of forward and reverse primers (with 0.5 μM concentration). Each run was proceeded in duplicate and further the average was considered as a single sample. The results were normalized according to the best reference gene, glyceraldehyde 3-phosphate dehydrogenase (GAPDH), and chosen from two other genes (β-actin, 18S ribosomal RNA) by the NormFinder software (Aarhus University, Denmark). The primer sequences are presented in Table 1.

**Table 1:** Oligonucleotide sequences used for Real-time PCR.

Gene name	Primers sequence (5′–3′)	Amplicon length (bp)	EMBL
*GAPDH*	F: CACCCTCAAGATTGTCAGCA	103	BC 102589
	R: GGTCATAAGTCCCTCCACGA		
*ACTB*	F: CCAAGGCCAACCGTGAGAAAAT	256	K00622
	R: CCACATTCCGTGAGGATCTTCA		
*RN18S1*	F: AAGTCTTTGGGTTCCGGG	365	AF176811
	R: GGACATCTAAGGGCATCACA		
3β-HSD	F:TGTCATTGACGTCAGGAATGC	100	NM_176644.2100
	R: TACGCTGGCCTGGACACA		
*P450*	F: TTGTGAACCAGTGGCAGATCAA	64	AF091667.1
	R: GGCCGGAACTCAGATGGAT		
*PR*	F: GGCAATTGGTTTGAGGCAAA	196	AJ557823.1
	R: TCTTGGGTAACTGTGCAGCAA		
*ERα*	F: ATGACCCTGCACACCAAAG	100	NM_001001443.1
	R: CCTCGGGGTAGTTGTACACG		
*17β-HSD*	F: TGTGCCCTCTCGGATTGTAG	244	AF265564
	R: AGTGACAGCCCTGACCAAAG		
*AKR1C1*	F: ATACAACGTGGGGTTGTGGT	126	S54973.1
	R: AGGACCATGATGGATTGCTC		

*GAPDH* Glyceraldehyde 3-phosphate dehydrogenase, *RN18S1* 18S ribosomal RNA, *ACTB* β-actin, *AKR1C1* aldo–keto reductase family 1 member C1, *3β-HSD* 3-beta-hydroxysteroid dehydrogenase, *17β-HSD* 17-beta-hydroxysteroid dehydrogenase, *P450* cytochrome P450 aromatase, *ESR1* estrogen receptor 1, *PR* progesterone receptor.

For efficiency evaluation, standard curves consisting of serial dilutions of the cDNA were plotted and the best cDNA concentration was chosen for further analysis. The first stage of the reaction was the initial denaturation of the strand and activation of the polymerase (95 °C for 2 min). The next stage consisted of 45 cycles of successive reactions: denaturation (95 °C for 5 s), primer annealing, and elongation of PCR products (6 °C for 20 s). To ensure the reaction’s specificity, the melting curves of the PCR products were analyzed after the amplification was completed. Data obtained were analysed using the Miner program. Control reactions lacking the template or primers were performed to confirm that products were free of primer-dimers and genomic DNA contamination, respectively. PCR products were sequenced and compared with the appropriate genes in NCBI.

### Total protein isolation

The uterine tissue (30 mg; separately endometrium and myometrium) was homogenized on ice in a RIPA lysis buffer (5 mM EDTA, 150 mM NaCl, 50 mM TRIS, 0.1% SDS, 1% Triton X-100, 0.5% sodium deoxycholate, and protease inhibitor-Sigma-Aldrich, S8830, pH 7.4). The obtained lysate was centrifuged at 10,000×*g* for 20 min at 4 °C, and the supernatant was transferred to a fresh tube and sonified. The protein concentration was estimated according to the Bradford’s method. The lysate was stored at − 80 °C until further analysis.

### Western blot analysis

The expression of P450, 17β-HSD, 3β-HSD, AKR1C1, PRs, and ERα proteins in the uterine tissues was determined by Western Blotting. For each sample, 30 µg of total protein was mixed with 5 µl of SDS gel-loading buffer, heated at 95 °C for 5 min, and separated in 10% SDS-PAGE. Afterward, the proteins were transferred on 0.2 μm nitrocellulose membranes in transfer buffer during the electroblotting for 1.5 h. The membranes were blocked in a 5% solution of skimmed milk with 1xTBS-T for 1.5 h in room temperature (RT), and then incubated overnight at 4 °C with specific primary antibodies for P450 (Abcam, Cambridge, UK, ab28146, 1:1000), 17β-HSD (Thermo Fisher Scientific, PA5-30063, 1:500), 3β-HSD (Gene Tex, Ca, USA, GTX114081, 1:500), AKR1C1 (Thermo Fisher Scientific, PA5-21672, 1:500), PRs (Thermo Fisher Scientific, MA1-410, 1:1000), and ERα (Thermo Fisher Scientific, MA1-310, 1:1000). As a reference the protein β-actin (ACTB, Sigma-Aldrich, A2228, 1:1000) was used. After that, the membranes was washes three times for 10 min in 1xTBS-T solution and incubated with secondary polyclonal anti-rabbit antibodies for P450, 3β-HSD, 17β-HSD, and AKR1C1 enzymes (Sigma-Aldrich, A3687, 1:10,000), and anti-mouse for PRs and ERα (Sigma-Aldrich, A3562, 1:10,000) for 1.5 h at RT. The protein bands were visualized using AP buffer and NBT-BCIP solution (Sigma-Aldrich, 72091). Western blots were quantitated using Quantity One 1-D Analysis Software (Bio-Rad, Hercules, CA, USA). Blots after transer are presented as a supplementary file.

### ELISA

Plasma concentrations of LH, FSH, T4, P4, and E2 after validation^25^ were evaluated using ELISA. LH and FSH concentrations were estimated using a kit for bovine (Cloud-Clone Corp., TX, USA, CEA441Bo for LH and CEA830Bo for FSH). The assay’s sensitivity for LH was 137.3 pg/ml and for FSH was 0.93 pg/ml. The mean intra- and inter- assays CVs were 10% and 12%, respectively. A commercial kit (Abbexa, abx574314) designed to measure the concentration of T4 has been assessed for measuring T4 in the plasma. The sensitivity of the assay was 1.95 pg/ml. The intra- and inter- assays CVs were 5.8 and 7.9%, respectively. According to the manufacturer’s instructions, the P4 and E2 concentrations were measured in plasma samples using an ELISA kit (Bioassay Technology Laboratory, Shanghai, China, E0018Bo for P4 and E0004Bo for E2). The assay’s sensitivities for P4 and E2 were 0.22 ng/ml and 2.53 pg/ml respectively. The mean intra- and inter -assays CVs were found to be 8% and 10%, respectively. Each run was proceeded in duplicate and further the average was considered as a single sample. All samples were detected in duplicate at 450 nm.

### Statistical analysis

GraphPad PRISM (Version 8.3.0, San Diego, CA, USA) was used for data analysis. The relationship between the mRNA and protein expression between endometrium and myometrium and between each experimental group throughout endometrium and myometrium was determined using two-way analysis of variance (ANOVA) followed by a Bonferroni post hoc test. One-way ANOVA was used for analysis of hormones` concentration, followed by Tukey’s test. All numerical data were expressed as the arithmetic mean ± standard error of mean (SEM). Statistical significance was *P* < 0.05.

## Supplementary Information

Supplementary Information.
