# Supplementary material for: Regulation of uterine function during estrous cycle, anestrus phase and pregnancy by steroids in red deer (Cervus elaphus L.)
Source: Sci Rep. 2021 Oct 11;11:20109. doi: 10.1038/s41598-021-99601-5 (PMC8505504; doi:10.1038/s41598-021-99601-5)
Supplement: Supplementary file 1 — Supplementary Information. [file 41598_2021_99601_MOESM1_ESM.docx]

**ACTB – 42 kDa**


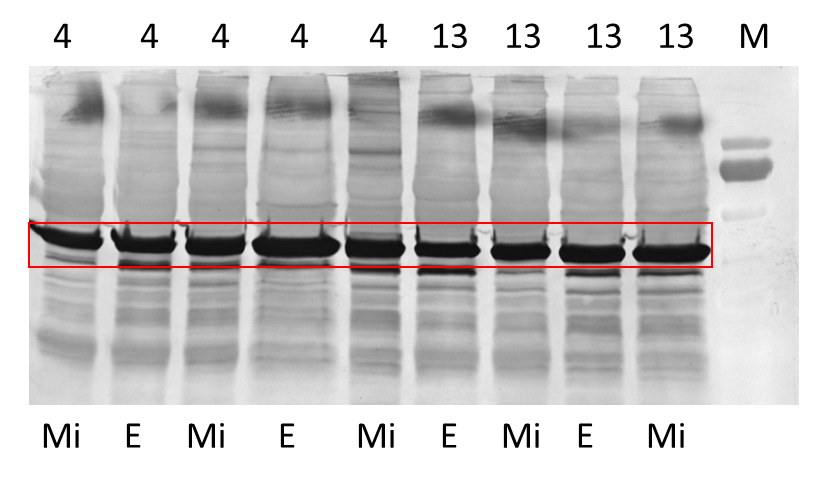


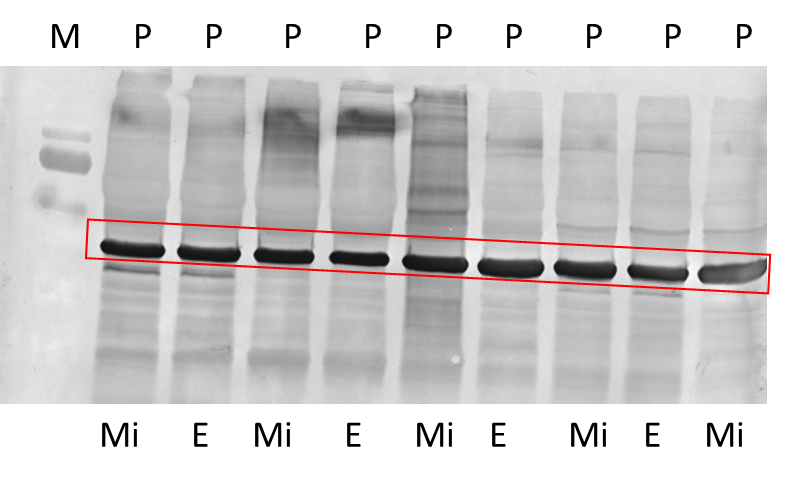


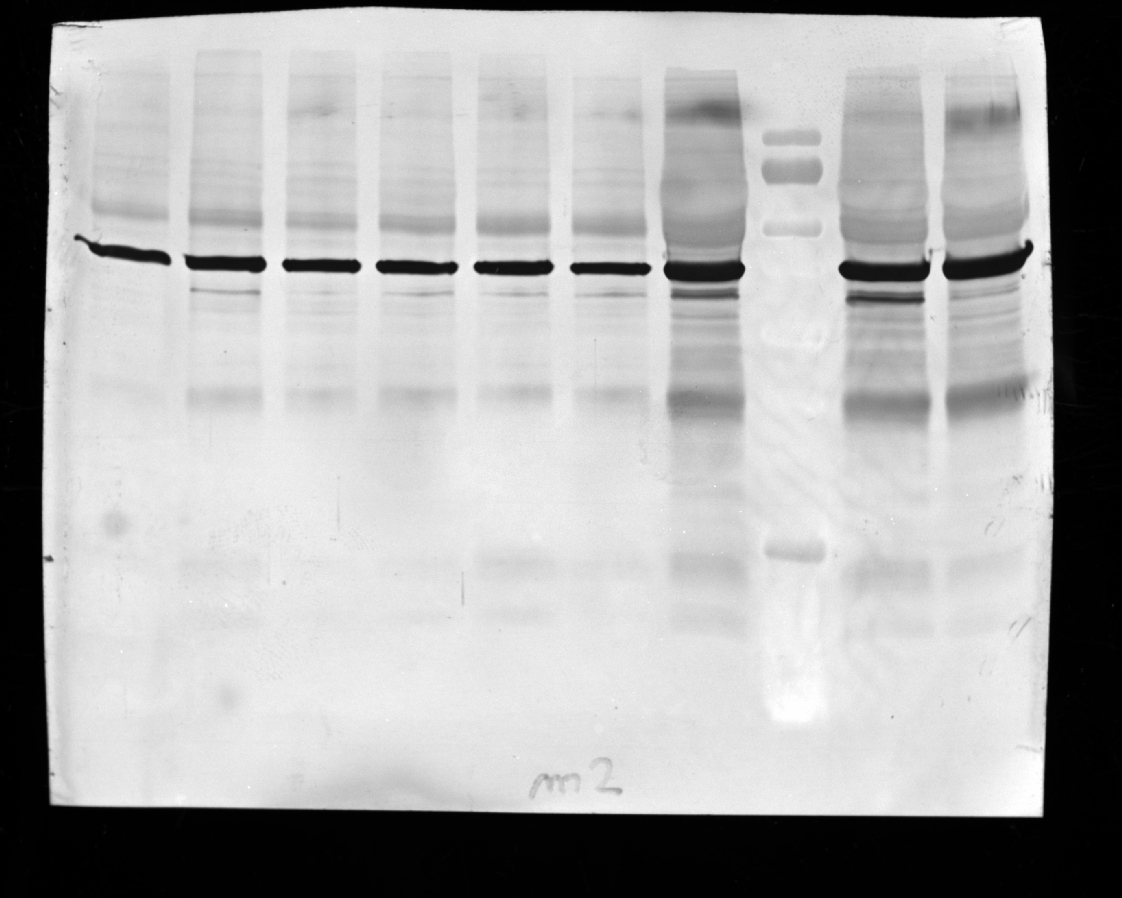


A A A A A A 13 M

Mi E Mi E Mi E Mi

Legend:

M – Marker,

13 – 13th day of the estrous cycle

4- 13th day of the estrous cycle

E- endometrium

Mi – myometrium

A-anestrus

**AKR1C1 – 37 kDa**


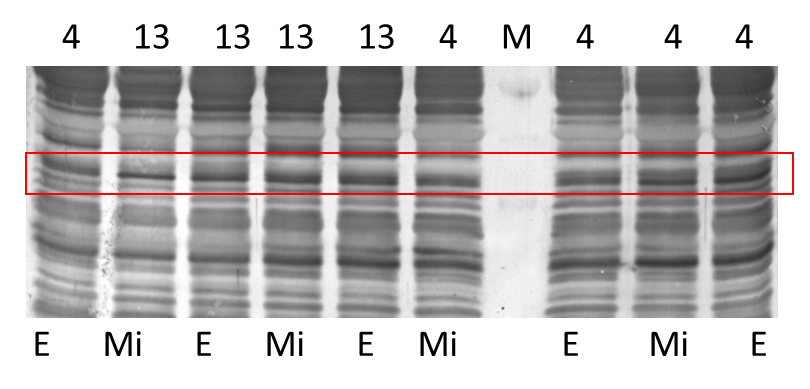


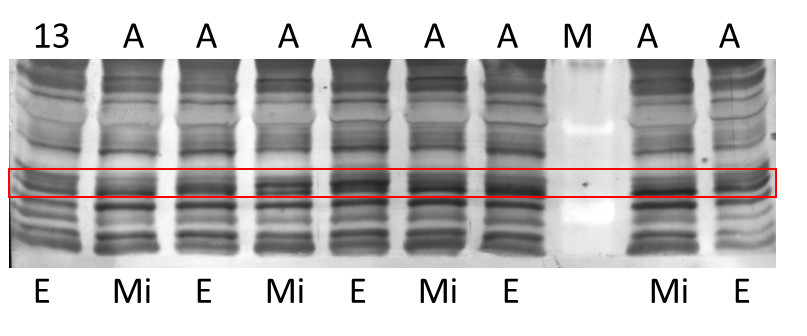


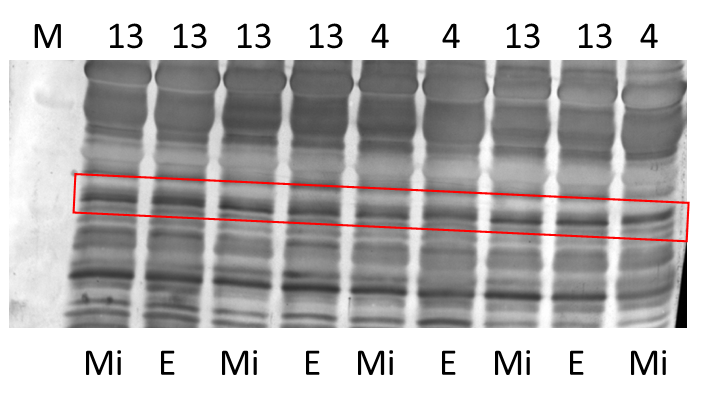


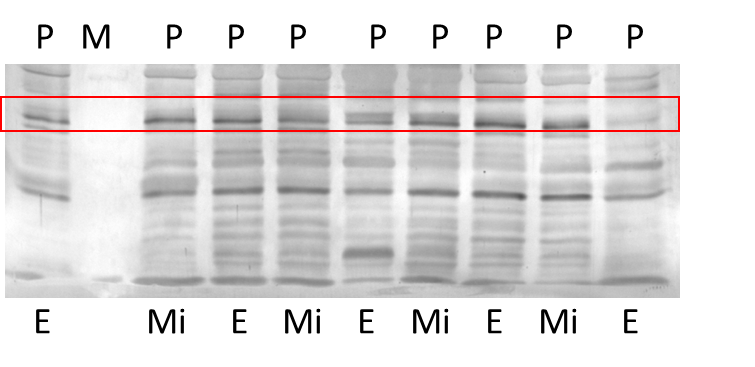


Legend:

M – Marker,

13 – 13th day of the estrous cycle

4- 13th day of the estrous cycle

E- endometrium

Mi – myometrium

A-anestrus

**PRs – 94-120 kDa**


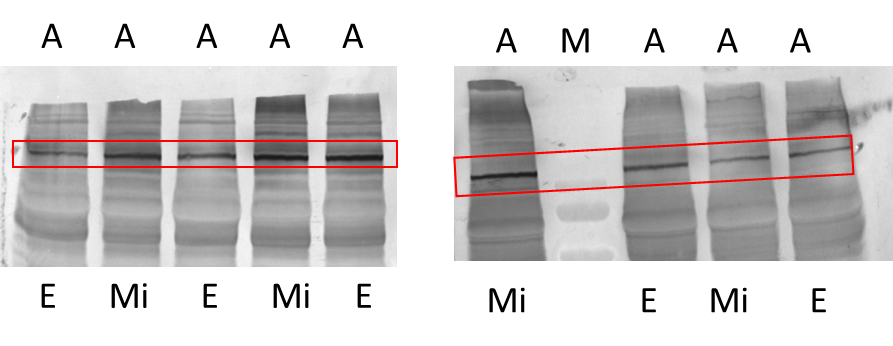

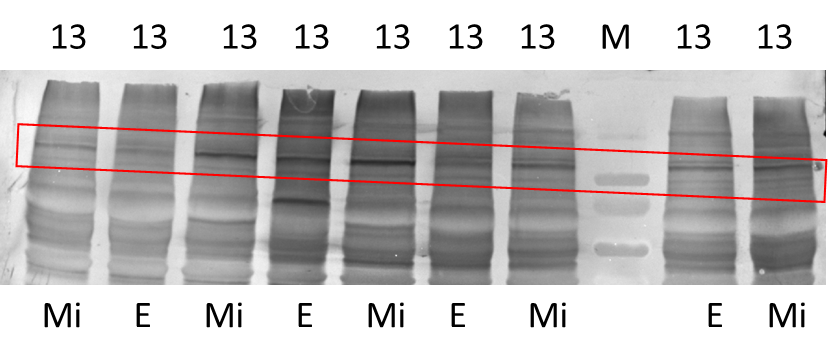

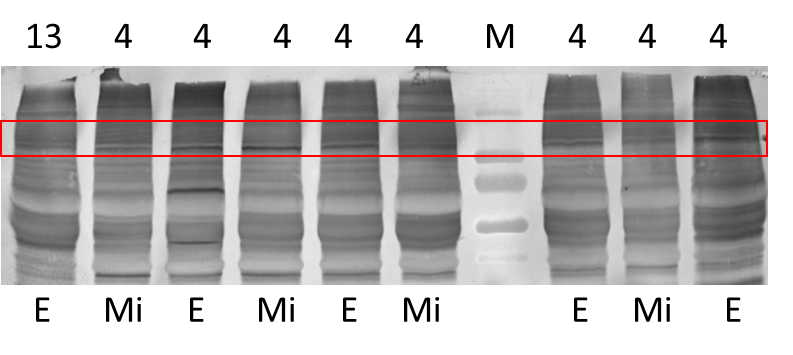


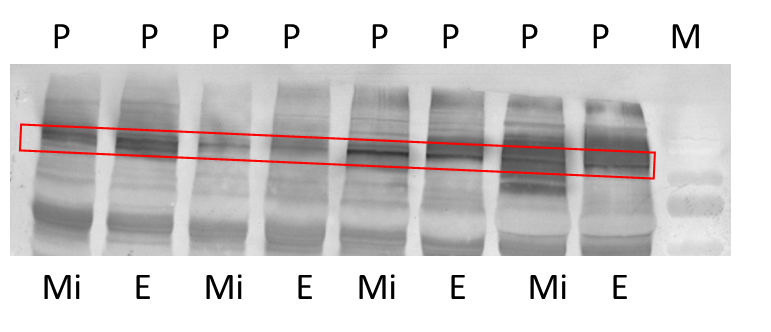


Legend:

M – Marker,

13 – 13th day of the estrous cycle

4- 13th day of the estrous cycle

E- endometrium

Mi - myometrium

A-anestrus

**ERα – 66 kDa**


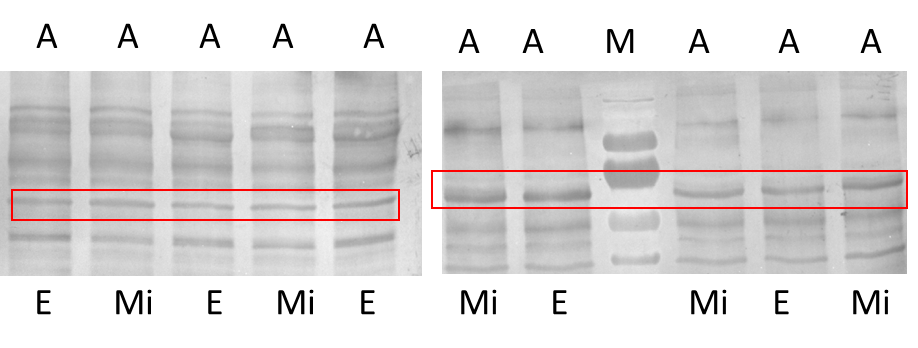

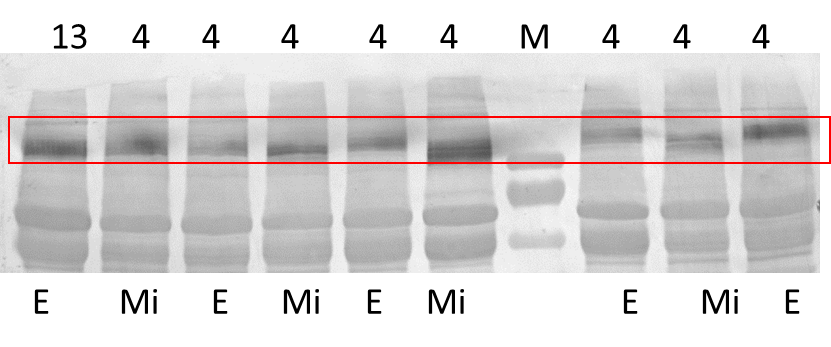


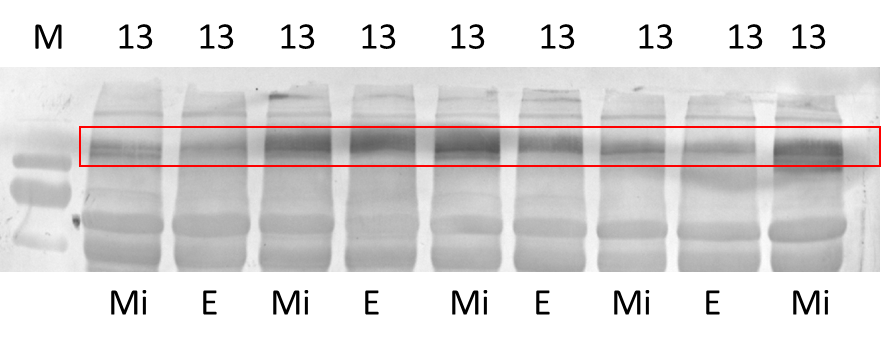


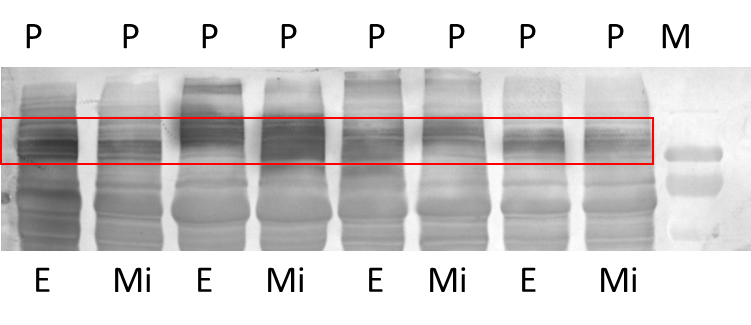


Legend:

M – Marker,

13 – 13th day of the estrous cycle

4- 13th day of the estrous cycle

E- endometrium

Mi – myometrium

A-anestrus

**P450 – 50-55kDa**


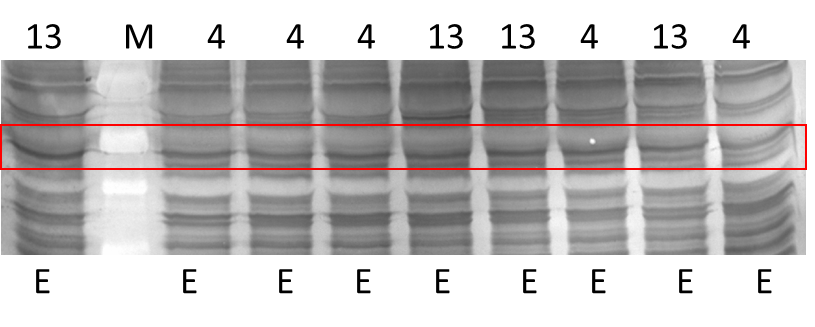


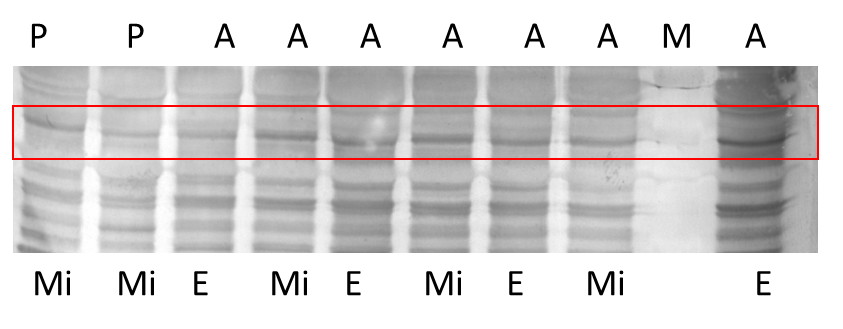

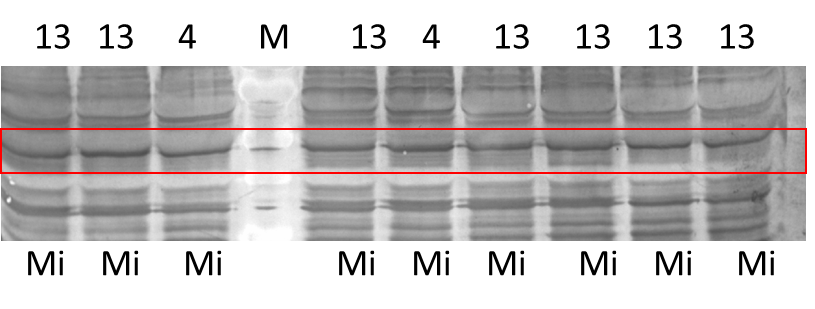


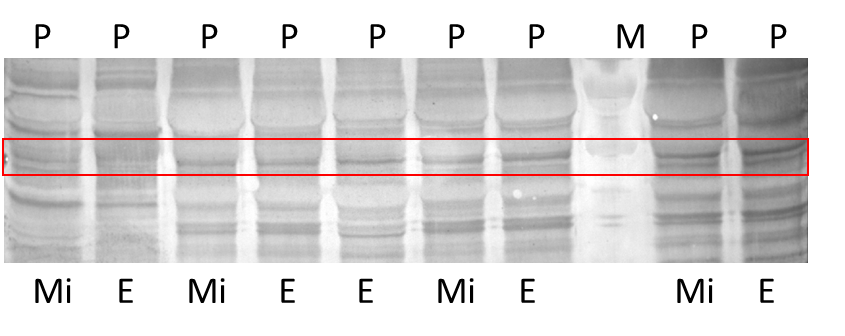


Legend:

M – Marker,

13 – 13th day of the estrous cycle

4- 13th day of the estrous cycle

E- endometrium

Mi – myometrium

A-anestrus

**17β- HSD – 32 kDa**


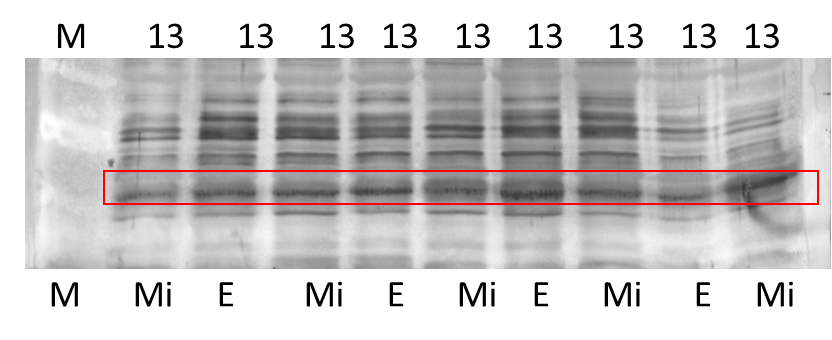


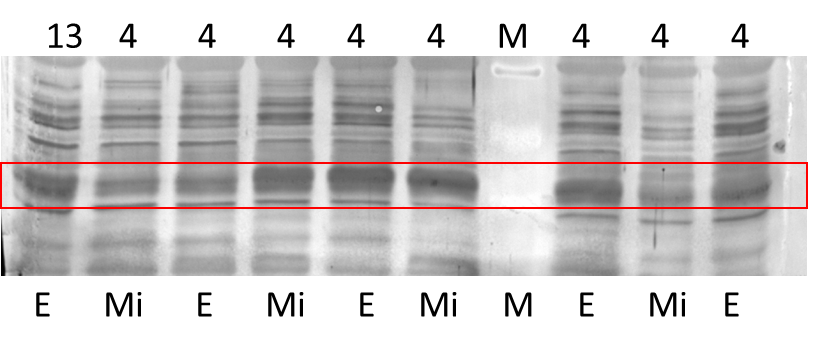


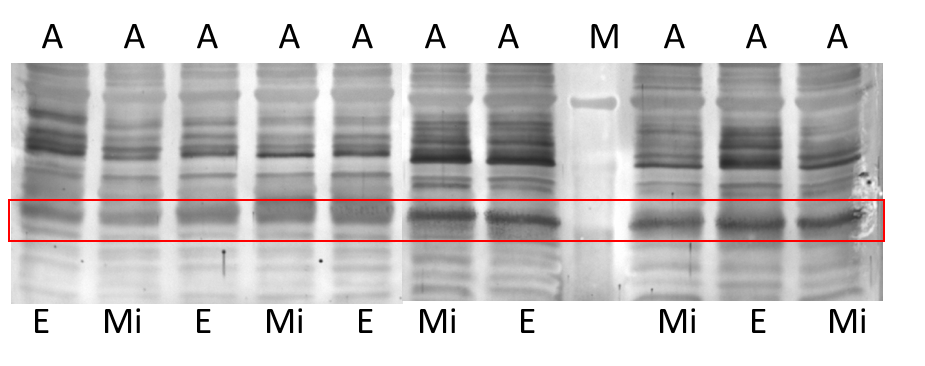


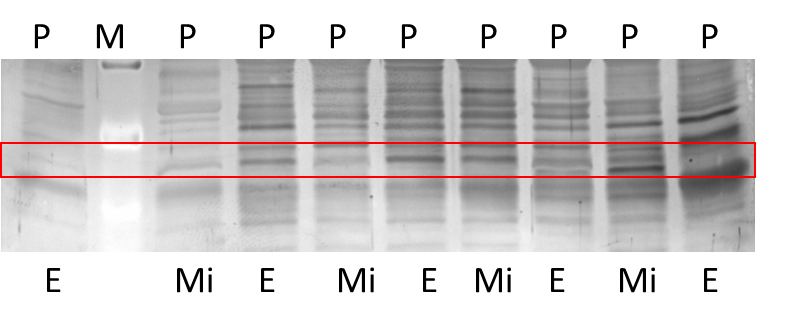


Legend:

M – Marker,

13 – 13th day of the estrous cycle

4- 13th day of the estrous cycle

E- endometrium

Mi – myometrium

A-anestrus

**3β- HSD – 40-42 kDa**


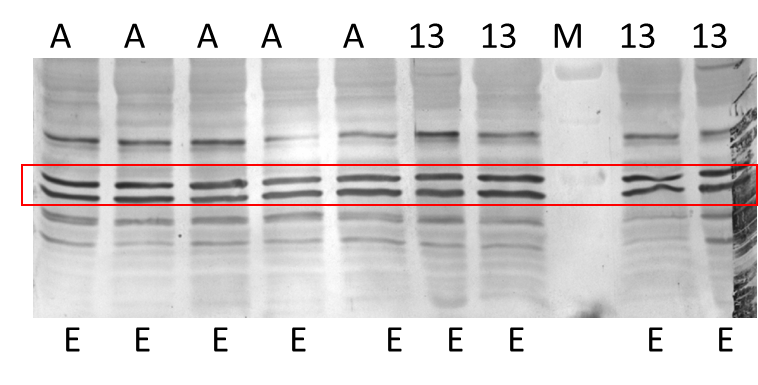


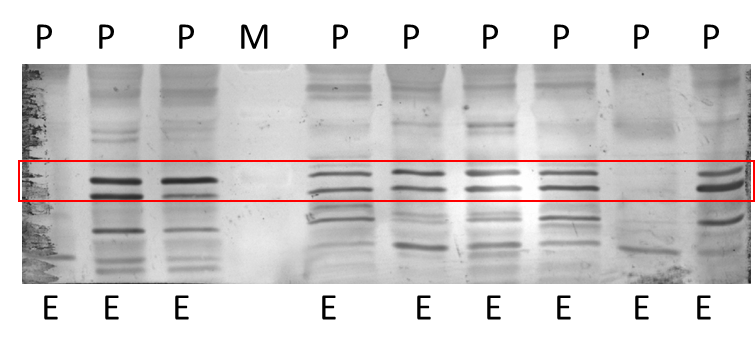

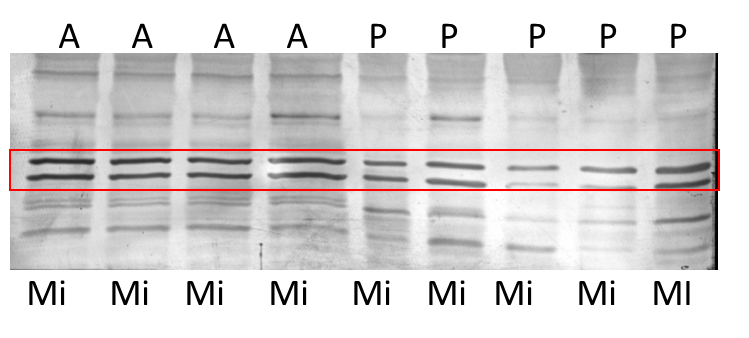


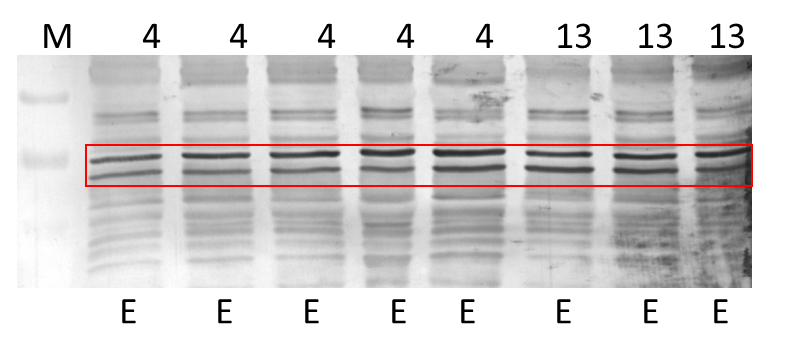


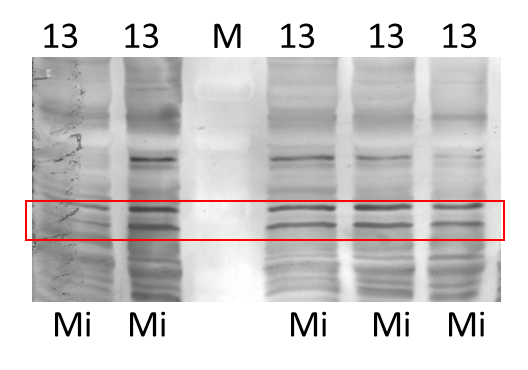

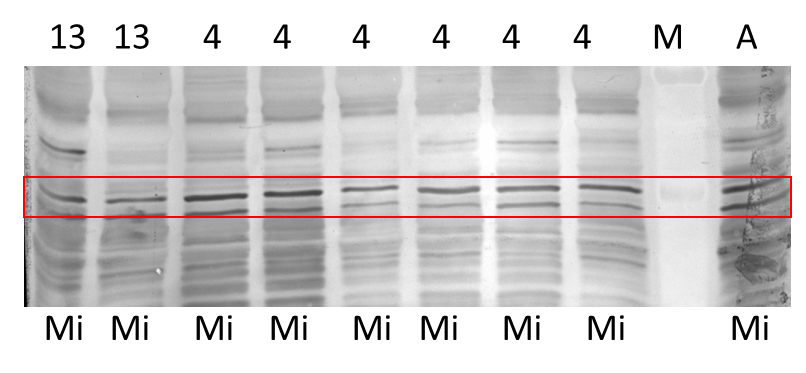


Legend:

M – Marker,

13 – 13th day of the estrous cycle

4- 13th day of the estrous cycle

E- endometrium

Mi – myometrium

A-anestrus
